# The assessment of routine health information system performance towards improvement of quality of reproductive, maternal, newborn, child and adolescent health services in Ondo and Ekiti States, Nigeria

**DOI:** 10.1371/journal.pone.0318010

**Published:** 2025-01-30

**Authors:** Victoria Oladoyin, Sunday Adedini, Kayode Ijadunola, Hassan Ogunwemimo, Olubunmi Folorunso, Elizabeth Chukwu, Ugo Okoli, Anthony Adoghe, Samuel Oyeniyi, Oniyire Adetiloye, Adesegun Fatusi

**Affiliations:** 1 Department of Community Medicine, Faculty of Clinical Sciences, University of Medical Sciences, Ondo City, Nigeria; 2 Department of Demography and Social Statistics, Faculty of Social Sciences, Federal University Oye Ekiti, Oye Ekiti, Nigeria; 3 Demography and Population Studies Programme, Schools of Public Health and Social Sciences, University of Witwatersrand, Johannesburg, South Africa; 4 Department of Community Health, Faculty of Clinical Sciences, Obafemi Awolowo University, Ile-Ife, Nigeria; 5 Elizade University, Ilara-Mokin, Nigeria; 6 Centre for Health Metrics and Data Science, School of Public Health, University of Medical Sciences, Ondo City, Nigeria; 7 Department of Human Nutrition and Dietetics, Faculty of Allied Health Sciences, University of Medical Sciences, Ondo City, Nigeria; 8 Jhpiego Nigeria, An affiliate of Johns Hopkins University, Baltimore, Maryland, United States of America; 9 Department of Planning Research and Statistics, Federal Ministry of Health, Abuja, Nigeria; 10 Department of Family Health, Federal Ministry of Health, Abuja, Nigeria; 11 Centre for Adolescent Health and Development, School of Public Health, University of Medical Sciences, Ondo City, Nigeria; University of Wyoming, UNITED STATES OF AMERICA

## Abstract

**Background:**

Nigeria’s reproductive, maternal, newborn, child, and adolescent health indicators have remained unsatisfactory in the face of poor-quality healthcare services. Nigeria initiated the reproductive, maternal, newborn, child, and adolescent, elderly + nutrition (RMNCAEH+N) quality of care (QoC) agenda to address the challenge. The health management information system (HMIS) is integral to the agenda but there is sparse evidence on its performance so far. This study assessed the performance of routine HMIS for RMNCAEH+N QoC in Ondo and Ekiti States.

**Methods:**

This paper described the review of health facility records and health facility survey components of a multi-component study which employed a mixed-method research design. Using the routine health information system performance diagnostic tool, service data captured for over one year were critically reviewed in randomly selected sample of 169 public health facilities (Ondo:117; Ekiti:52) and information was obtained from facility heads or designates. Performance of routine HMIS for RMNCAEH+N QoC in terms of data collection, data quality, and data use were analysed using univariate and bivariate statistics.

**Results:**

Results show that 67.3% of health facilities in Ekiti and 88.9% of facilities in Ondo had all required HMIS tools for selected RMNCAEH+N services (p<0.001). Data accuracy was 70.1% for Ondo and 40.4% for Ekiti (p <0.001); 82.9% of facilities in Ondo and 44.2% in Ekiti had complete data (p <0.001); almost all facilities (Ondo: 99.1%; Ekiti: 96.2%, p = 0.224) demonstrated data consistency; and, 82.9% of facilities in Ondo and 94.2% of facilities in Ekiti demonstrated timeliness in data submission (p = 0.048). Also, 70.1% (Ondo) and 78% (Ekiti) of facilities had quality improvement (QI) teams (p = 0.338); 53.5% (Ondo) and 77.1% (Ekiti) of QI teams regularly extracted data, calculated, and visualised prioritized indicators (p = 0.007); while 72.1% (Ondo) and 79.2% (Ekiti) regularly reviewed data and used it to make QI decisions (p = 0.367).

**Conclusion:**

Routine RMNCAEH+N QoC data management system in Ondo and Ekiti States vary in terms of the status of reporting forms, data quality, and data use for decision-making, and there were specific performance gaps. The routine RMNCAEH+N QoC data management system in Ondo and Ekiti States needs improvement and findings from this study can serve as the basis for evidence-based advocacy for the required efforts and investment toward improved performance.

## Introduction

Nigeria’s reproductive, maternal, newborn and child health (RMNCH) indicators have remained considerably poor over the years despite several laudable initiatives and efforts to improve the situation. Nigeria has the highest maternal mortality burden in the world: with approximately 82,000 maternal deaths, Nigeria accounted for more than a quarter (28.5%) of all estimated global maternal deaths in 2020 [1]. Nigeria does not only have the highest estimated maternal deaths, the gap in the absolute number of maternal deaths between Nigeria and the other three countries with estimated deaths of 10,000 and above is so wide with India, Democratic Republic of Congo (DRC), and Ethiopia having estimated maternal deaths of 24,000, 22,000, and 10,000 respectively [1]. Again, with an estimated 835,000 under-five deaths, Nigeria had the highest burden of under-five deaths in the world in 2022 and accounted for 17% of the 4.89 million under-five deaths globally [2] There is also a wide disparity between the under-five deaths reported in Nigeria and five other countries with under-five deaths of 100,00 and above: India (670,000), Pakistan (386,000), DRC (302,000), Ethiopia (178,000), and Niger (131,000) [2]. The RMNCH indicators for Nigeria have significant sub-national and socio-demographic variations, with the indices considerably poorer in some states/regions than others, and among some population groups compared to others [3].

Poor quality of care (QoC) is considered an important underlying contributor to the poor RMNCH indices recorded for Nigeria [4]. Nigeria’s public health system, which serves as the major provider of RMNCH services, is a critical hotspot for the poor quality of care [3, 5]. The World Health Organization (WHO) framework for the quality of maternal and newborn health care encompasses eight domains, including actionable health system; evidence-based practices for routine care and management of complications; and functional referral systems–these three domains relate to the provision of care. The other QoC domains include competent, motivated human resources; availability of essential physical resources; and three domains relating to the experience of care (effective communication; respect and preservation of dignity; and emotional support) [6]. A significant proportion of public-sector facilities in Nigeria have been reported to perform poorly in many of the QoC domains regarding RMNCH services [7–9].

An actionable health management information system (HMIS) plays a central role in understanding the health system performance, monitoring of QoC performance, and tracking the effect of quality improvement (QI) efforts as well as in decision-making and policies. Also, research evidence has shown that healthcare policymakers in Nigeria regard the routine health information system (RHIS) as the main source of evidence for decision-making at all levels of the health system, particularly health facility and local government area (LGA) levels [10]. The functioning and quality of performance of the HMIS are, thus, integral to QI initiatives and every effort to improve the quality of the health system performance at primary, secondary and tertiary levels. As part of the effort to improve RMNCH performance and with emphasis on quality of care, Nigeria developed its Integrated National Reproductive, Maternal, Child, and Adolescent Health (RMNCAH) + Nutrition Quality of Care Strategy in 2018 [11]. Subsequently, Nigeria integrated Elderly Health into RMNCAH to evolve its reproductive, maternal, newborn, child, adolescent, and elderly health plus nutrition (RMNCAEH+N) agenda and launched the RMNCAEH+N QoC operational plan [12] and the RMNCAEH+N QoC Monitoring, Evaluation, Accountability and Learning (MEAL) Plan [13].

This study is part of the national effort to assess progress in Nigeria’s strides towards RMNCAEH+N QoC and focuses specifically on the HMIS component in line with the emphasis of the MEAL Plan that “consideration of QoC data systems and measurements indicate the increased demand for quality data for decision making across all sectors [13].” This study aimed to assess the performance of the routine collection and use of RMNCAEH+N QoC data for decision-making at all levels of health care in Ondo and Ekiti States of Nigeria, in other to generate evidence for the strengthening of the RMNCAEH+N QoC information system for sustained high-quality performance at all levels of care. This paper, based on a multi-component study, focused specifically on the performance of the routine health information system regarding reproductive, maternal, newborn, child, and adolescent health services in the context of quality of care. The study assessed the HMIS performance in two neighbouring southwestern Nigerian states–Ondo and Ekiti States–in terms of availability of relevant data collection materials; data accuracy, completeness, consistency, and timeliness in submission to the required authorities; and data use.

## Materials and methods

### Study design and participants

This cross-sectional study was conducted in Ekiti and Ondo States, which are two neighbouring states in southwest Nigeria. The two states are historically linked in terms of culture, socio-demographic trends, and political and economic developments. The states are domiciled by the same Yoruba ethnic group, and both states were part of the old western region and together constituted the old Ondo State. As the 2018 Nigeria Demographic and Health Survey shows, Ondo and Ekiti States have the worst RMNCH indices among the six states that constitute the present south-west geo-political zone of Nigeria. For example, for the south-western states, the under-5 mortality rate was 30 deaths per 1000 live births in Ogun State compared with 79/1,000 for Ondo State and 95/1,000 for Ekiti State [3]. Similar patterns were obtained for other key RMNCH indicators. Overall, the study consists of four related components and employed a mixed-method implementation research design: (i) review and analysis of the routine health information system QoC records for RMNCAEH+N at different levels of care (primary, secondary and tertiary); (ii) health facility survey involving primary quantitative data collection from heads of health facilities; (iii) LGA survey involving primary quantitative data collection from LGA HMIS officers at the LGA offices; and, (iv) qualitative data collection with diverse stakeholders, including health workers, clients, and clients’ relations who accompanied them to the health facility for services. This paper focuses on the first and second components of the study–the review of facility-based HMIS registers that covered, among others, the HMIS daily registers and health facility monthly summary form records for the one-year preceding the survey; and structured consultations with the Officers-in-Charge of PHCs and Medical Directors of general and teaching hospitals or their designated representatives. Data collection for this component of the study was carried out over three weeks from November 2 to November 23, 2023 and covered public health sector facilities across the three categories of health care facilities in the Nigerian health system–primary, secondary, and tertiary health care facilities.

Ethical approvals were obtained for the study from the following the Research Ethics Committee/Boards of the following institutions: University of Medical Sciences, Ondo State (NHREC/TR/UNIMED-HREC-Ondo St/22/06/21; 22^nd^ March 2023); Ondo State Ministry of Health (OSHREC 20/03/23/528); Ekiti State University Teaching Hospital (EKSUTH/A67/2023/03/015); Ekiti State Ministry of Health and Human Services (MOH/PRS/158/44); and Johns Hopkins University Institutional Review Board (IRB#:23772). Permissions were also appropriately collected from relevant government agencies (State Hospital Management Boards and the State Primary Health Care Board/Agency) and heads of health facilities in both Ondo and Ekiti States before the data collection.

We determined the number of facilities to be involved in the study as 165, based on the use of the sample size formula for estimating single proportions and the figure of 87.8% as the proportion of health facilities documented by the DHIS2 database of the Nigerian Federal Ministry of Health as reporting 100% of key RMNCAEH-related service indicators for the two states. With Ondo State responsible for 70% of the total number of reporting facilities compared to Ekiti State’s 30%, we applied the principle of probability-proportionate-to-size to arrive at a minimum sample size of 115 facilities for Ondo State and 50 facilities for Ekiti State. Our focus was public health sector providing a mix of RMNCAEH services, including maternal health care services such as family planning (FP), antenatal care (ANC), labour and delivery, and postnatal care (PNC) care; newborn and child health care services, including child growth monitoring, and immunisation services; and, adolescent health etc. To be included in the study the facility must meet two other criteria: (i) provision of at least a combination of two of the RMNCAEH+N target services; and (ii) evidence of submission of at least one report on RMNCAEH+N services of interest on the DHIS2 platform of the Federal Ministry of Health in the last one year preceding the survey.

Sampling of the health facilities was done using a multistage sampling technique. Two senatorial districts, out of three senatorial districts in each state were selected. The senatorial districts left out in the two states were those with high-security concerns at the time of the study, which made them unsafe for data-collecting activities. In the second stage of sampling, nine and six Local Government Areas (LGAs) were selected from Ondo and Ekiti States respectively using simple random sampling technique. Lastly, using simple random sampling technique, we selected thirteen health facilities from each of the nine selected LGAs in Ondo State and targeted nine health facilities from each of the six selected LGAs in Ekiti State. However, one of the LGAs in Ekiti State had only seven health facilities, and all were included in the study. Overall, 169 public-sector health facilities offering at least two components of RMNCAEH-related services were selected, consisting of 117 facilities from Ondo State and 52 facilities from Ekiti State.

Data collection was carried out using electronic tablets and phones. The data collection instrument was adapted from the “Performance of Routine Information System Management” (PRISM) toolkit developed by MEASURE Evaluation [14]. The tool was modified in line with the objectives of the study and pretested in health facilities in a neighbouring south-western state (Osun State), and then scripted on mobile surveyCTO software. The data collection targeted the head of the health facility (Officers-in-Charge in case of Primary Health Care facilities, Medical Directors in the case of Secondary Health Care facilities, and Chief Medical Directors in the case of Tertiary Health Care facilities) or their designated representatives, and HMIS Officers. Strict quality assurance processes and oversight were maintained during the data collection to ensure quality data collection using a combination of highly experienced field supervisors and control quality officers who manned the backend of the data submission processes on a synchronous and continuous basis.

### Variables measurement and data management

The variables measured were in three categories (Table 1): (i) Availability of standardized registers and data collection forms for selected RMNCAEH+N services, and procedure manual/guide/standard operating procedure (SOP) for data collection; (ii) Quality of the data collected at primary, secondary, and tertiary healthcare levels in terms of accuracy, completeness, consistency, and timeliness. Data accuracy was based on comparison of data collected and entered into the daily facility register with the data submitted to the stipulated administrative level at two different points (August, 2022 and February, 2023). Data completeness was based on the data in the monthly record for June 2023. Data consistency was measured by reviewing the national HMIS health facility monthly summary form (NHMIS/HF/MSF) to see if the same RMNCAEH service items were captured every month from July 2022 to June 2023. Timeliness was assessed by comparing the time the records were submitted to the next administrative level over the one year of focus compared to when they were expected to have been submitted based on the protocol of Nigeria’s National Health Information System (NHMIS). The specific RMNACEH+N services or service elements that the different aspects of the data quality assessment focused on are included in Table 1 along with the indicators of interest that were sourced from the national RMNCAEH+N MEAL Plan; (iii) Existence and functioning of QI teams, including regular extraction of data, calculation and displaying prioritized quality indicators in the health facility, and using data for decision-making.

**Table 1:** Variable measurements: Domains, elements and indicators for HMIS QoC performance assessment.

Elements of Quality Measurement (by Domains)	Indicators
***Domain I*: *Availability of standardized registers and data collection forms and procedure manual/guide/standard operating procedure (SOP) for data collection***	
A) Standardized registers and data collection forms for specific services	
*(a) General patient management*	% facilities with standardized registers and data collection form for each service % facilities with standardized registers and data collection form for all the 9 specified services regarding general patient management, maternal health care and child health care
(i) Daily general facility attendance register	
(ii) Daily outpatient register	
(iii) NHMIS/health facility/monthly summary form	
*(b) Maternal health care*	
(iv) Family planning (FP) register	
(v) Antenatal care (ANC) register	
(vi) Labor and delivery register	
(vii) Postnatal care (PNC) register	
*(c) Child health services*	
(viii) Immunization register	
(ix) Growth monitoring and promotion register	
B) Availability of procedure manual/guide/standard operating procedure (SOP) for data collection	% facilities with procedure manual/guide/standard operating procedure (SOP) for data collection
***Domain II*: *Quality of the data collected in terms of completeness*, *accuracy*, *consistency*, *and timeliness***	
A). Data accuracy Comparison of data in the daily register with the submission made to the central level for the following services (and months)	
(i) Penta 3 vaccine (August 2022)	% facilities with accurate records for each service element % facilities with accurate records for all the 10 service elements (overall data accuracy)
(ii) Penta 3 vaccine (February 2023)	
(iii) 4^th^ ANC visit (August 2022)	
(iv) 4^th^ ANC visit (February 2023)	
(v) Total deliveries (August 2022)	
(vi) Total deliveries (February 2023)	
(vii) Babies 0–6 months exclusively breastfed (August 2022)	
(viii) Babies 0–6 months exclusively breastfed (February 2023)	
(ix) Children 6–59 months who received vitamin A supplementation (August 2022)	
(x) Children 6–59 months who received vitamin A supplementation (February 2023)	
B: Data completeness, with focus on:	
Maternal health care: family planning, antenatal care, delivery, postnatal care	% facilities with data completeness for each of the 14 service elements across maternal health, newborn and child health, school and adolescent health, and others/treatment of disease conditions
Newborn and child health: newborn health, childhood immunization, growth monitoring and promotion, child health and integrated management of childhood illnesses, and nutrition rehabilitation	
School and adolescent health: school health, adolescent health	
Others/treatment of disease conditions: adult outpatient, malaria treatment, treatment of sexually transmitted infections	
C. Consistency of RMNCAEH+N data collected at the health facilities Facilities with the same RMNCAEH service data captured every month from July 2022 to June 2023 in the NHMIS/HF/MSF	% of facilities with consistent data
D. Timeliness of RMNCAEH+N data collected at the health facilities Facilities where monthly reports are sent to the LGA office on the expected dates every month	% of facilities with timely submission
**Domain III: Existence and functioning of Quality Improvement (QI) team**	
A. Existence of QI teams in the health facility	% facilities with QI teams in place
B. QI team regularly extracts data, calculates, and visualizes prioritized quality indicators	% facilities in which QI team regularly extracts data, calculates, and visualizes prioritized quality indicators
C. QI team regularly review and use data to make decisions on quality-of-service issues	% facilities where data is regularly reviewed and used to make decisions on QI

Data analysis was undertaken through the use of Stata statistical software (version 14.2) and relevant indicators were generated for the two states individually (univariate analysis). Bivariate analysis (Chi-square test) was calculated to assess if significant difference existed in the performance of the two states on key data issue. Level of statistical significance was set at 5%.

### Ethical consideration

As noted earlier, ethical approvals and relevant administrative permissions were obtained from relevant institutions, agencies, and responsible persons across various health care levels in the two states before the study and data collection processes. Data collectors were trained on research ethics and monitored for adherence by supervisors during the fieldwork. Written informed consent was obtained from each respondent before the administration of the study tool. The study was conducted with adherence to highest ethical standards.

Furthermore, an interactive data validation and dissemination meeting was organised in each state and attended by the heads of the State Ministry of Health (the Commissioner of Health and Permanent Secretary of the Ministry of Health in Ekiti and Ondo State respectively), heads of the State Hospital Management Boards and Primary Healthcare Board/Agency, Medical Officers of Health in charge of the local government areas where the study was collected, and head of the various healthcare facilities where data was collected. The dissemination meeting involved discussions by the stakeholders on the implications of the results for quality health care and actions needed to address deficiencies and improve the quality of health services.

## Results

Of the 169 health facilities surveyed, 117 (69.2%) were based in Ondo State and 52 (30.8%) in Ekiti State. One hundred and fifty-one of the facilities (89.3%) were primary health care (PHC) facilities, while fifteen (8.9%) were secondary health care (SHC) facilities, and three (1.8%) were tertiary health care (THC) facilities. One hundred and three of the respondents (61.0%) were head of facilities—95 PHC officers-in-charge, 5 SHC medical directors, 3 THC chief medical directors–while twelve (7.1%) were HMIS officers and others (32.0%) were representative of the heads of health facilities.

### Availability of standardized registers and data collection forms for selected RMNCAEH+N services

As Table 2 shows, general facility attendance register and daily outpatient register were available in virtually all the health facilities in both states (at least 99% of facilities), and less than 5% of the facilities that had these registers available experienced any stock out of those registers in the 12 months preceding the study. However, while over 95% (95.7% for Ondo and 98.1% for Ekiti) had the health facility monthly summary form, more than two-fifths of the facilities that had the form experienced stock out at least once within the preceding 12-month period (42.9% for Ondo and 47.1% for Ekiti).

**Table 2:** Availability of RMNCAEH+N registers/forms and stock-out in the 12 months preceding the survey.

Registers/forms	Ondo State		Ekiti State	
	Availability of form/ register (N = 117)	Stock-out experienced	Availability of form/ register (N = 52)	Stock-out experienced
	Yes (%)	Yes (%)	Yes (%)	Yes (%)
***General patient care***				
Daily general facility attendance register	117 (100)	4 (3.4)	52 (100)	2 (3.8)
Daily outpatient register	116 (99.1)	3 (2.6)	52 (100)	1 (1.9)
Health facility monthly summary form	112 (95.7)	48 (42.9)	51 (98.1)	24 (47.1)
***Maternal health care***				
Family planning (FP) register	117 (100)	0 (0)	49 (94.2)	0 (0)
Antenatal care (ANC) register	116 (99.1)	0 (0)	46 (88.5)	0 (0)
Labor and delivery register	115 (98.3)	0 (0)	45 (86.5)	0 (0)
Postnatal care (PNC) register	116 (99.1)	0 (0)	40 (76.9)	0 (0)
***Child health***				
Immunization register	114 (97.4)	1 (0.9)	49 (94.2)	0 (0)
Growth monitoring and promotion register	111 (94.9)	14 (12.6)	45(86.5)	1(2.2)

The availability of registers for maternal health services ranged from 98.3% (labour and delivery services) to 100% (family planning services) for health facilities in Ondo State but the availability in Ekiti State health facilities was considerably lower, ranging from 76.9% (PNC services) to 94.2% (family planning services); none of the facilities which reported availability of the listed maternal health care registers recorded stock-out of any of the maternal health registers within the last 12 months to the study. The child health service registers were also more available in Ondo State compared to Ekiti State and for immunisation (97.4% for Ondo State and 94.2% for Ekiti State) compared to growth monitoring (94.9% for Ondo State and 86.5% for Ekiti State). Stock out of the available child health registers was rarely experienced for immunisation register in both states, but 12.6% of the facilities in Ondo State and 2.2% of the facilities in Ekiti State experienced stock out for growth monitoring register within the 12 months prior to the study. This is also shown in Table 2.

Overall, the proportion of health facilities that had all the required tools for general services, maternal health services, and child health services was 88.9% for Ondo State and 67.3% in Ekiti State (p <0.001). Procedure manual/guide/standard operating procedure (SOP) for data collection was available in 86.5% of health facilities in Ekiti State compared to 75.2% of facilities in Ondo States (p = 0.098). These are shown in Table 3.

**Table 3:** Comparison of availability of data collection tools between states.

	Ondo State n (%)	Ekiti State n (%)	p-value
Availability of all general, maternal and child health services registers/forms			
No	13 (11.1)	17 (32.7)	
Yes	104 (88.9)	35 (67.3)	<0.001
Existence of a procedure manual/guide/SOP for data collection			
No	29 (24.8)	7 (13.5)	
Yes	88 (75.2)	45 (86.5)	0.098

### *Data accuracy and completeness*, consistency, and timeliness in submission

As Table 4 shows, the accuracy for the RMCAEH+N data collected by the health facilities ranged from 88.0% to 100% for Ondo State for various services compared to 57.7% to 94.2% for Ekiti State. The highest level of data accuracy for Ondo State was with respect to delivery records—99.1% for August 2022 data, and 100% for February 2023 data, while the lowest recorded accuracy was in respect of children (6–59 months) who received vitamin A supplementation: 90.6% for August 2022, and 88.0% for February 2023. The highest level of data accuracy for Ekiti State was with respect to 4^th^ ANC visit for August 2022 (94.2%), while the lowest recorded accuracy was in respect of babies (0–6 months) who were exclusively breastfed (57.7% for February 2023 data). Ondo State recorded a higher level of accuracy for each of the 10 RMNCAEH+N service of interest compared to Ekiti State. At least 90% of health facilities in Ondo State had data accuracy for nine of the 10 service elements considered in this study compared to only three service elements in Ekiti State with the same status.

**Table 4:** Accuracy of RMNCAEH+N data collected at the health facilities.

RNCAEH+N data	Ondo State (N = 117)		Ekiti State (N = 52)	
	Accurate reporting Freq (%)	Inaccurate reporting Freq (%)	Accurate Reporting Freq (%)	Inaccurate reporting Freq (%)
(i) Penta 3 vaccine (August 2022)	109 (93.2)	8 (6.9)	39 (75.0)	13 (25.0)
(ii) Penta 3 vaccine (February 2023)	111 (94.9)	6 (5.2)	48 (92.3)	4 (7.7)
(iii) 4^th^ ANC visit (August 2022)	112 (95.7)	5 (4.3)	49 (94.2)	3 (5.8)
(iv) 4^th^ ANC visit (February 2023)	109 (93.2)	8 (6.9)	46 (88.5)	6 (11.6)
(v) Total deliveries (August 2022)	116 (99.1)	1 (0.9)	45 (86.5)	7 (13.5)
(vi) Total deliveries (February 2023)	117 (100)	0 (0.0)	48 (92.3)	4 (7.7)
(vii) Babies 0–6 months exclusively breastfed (August 2022)	107 (91.5)	10 (8.5)	32 (61.5)	20 (38.4)
(viii) Babies 0–6 months exclusively breastfed (February 2023)	112 (95.7)	5 (4.3)	30 (57.7)	22 (42.3)
(ix) Children 6–59 months who received vitamin A supplementation (August 2022)	106 (90.6)	11 (9.4)	33 (63.5)	19 (36.5)
(x) Children 6–59 months who received vitamin A supplementation (February 2023)	103 (88.0)	14 (11.9)	35 (67.3)	17 (32.7)

For the individual service elements, the closest gap between the two states was in respect of the August 2022 record for ANC (95.7% versus 94.2%) and the February 2023 record for Penta 3 vaccine (94.9% versus 92.3%). On the other hand, the widest gap in the two states in terms of data accuracy was with respect to babies (0–6 months) who were exclusively breastfed: 91.5% versus 61.5% for August 2022, and 95.7% versus 57.7% for February 2023. Outside the data for Ekiti State regarding babies 0–6 months exclusively breastfed (57.7%–61.5%) and children 6–59 months who received vitamin A supplementation (63.5%–67.3%) for Ekiti State, at least three-quarters of the facilities in both Ondo and Ekiti States demonstrated data accuracy for the various service elements (Table 4).

Ondo State recorded a better performance than Ekiti State in terms of the completeness of RMNCAEH+N data reporting by health facilities across 10 of the 14 service elements covered in the study, cutting across maternal health, child health, and adolescent health, with adult outpatient service (100% vs. 99.1%) and sexually transmitted infection management (62.5% versus 60%) as the exceptions where Ekiti had a slightly better performance. In general, Ondo State had 100% of its facilities achieving data completeness regarding malaria treatment, while Ekiti State had a similar achievement with respect to malaria treatment and adult outpatient (Table 5).

**Table 5:** Completeness of RMNCAEH+N data collected at the health facilities.

RMNCAEH+N Services	Ondo State		Ekiti State	
	Facilities providing RMNCAEH+N services	Number of facilities with complete reporting Freq (%)	Facilities providing RMNCAEH+N services	Number of facilities with complete reporting Freq (%)
***Maternal health care***				
Family planning	115	113 (98.3)	47	44 (93.6)
Ante-natal care	117	116 (99.1)	49	43 (87.8)
Labour and delivery	117	114 (97.4)	48	41 (85.4)
Post natal care	116	115 (99.1)	47	38 (80.9)
***Newborn and child health services***				
Newborn health	117	111 (94.9)	48	40 (83.3)
Childhood immunization	117	114 (97.4)	49	47 (95.9)
Growth monitoring and promotion	116	113 (97.4)	47	40 (85.1)
Child health and integrated management of childhood illnesses	117	113 (96.6)	50	40 (80.0)
Nutrition rehabilitation	14	11 (78.6)	17	13 (76.5)
***School and adolescent health***				
School health	2	1 (50.0)	4	2 (50.0)
Adolescent health	5	3 (60.0)	6	3 (50.0)
***Treatment of disease conditions***				
Malaria treatment	116	116 (100.0)	51	51 (100.0)
Sexually Transmitted Infection Management	5	3 (60.0)	8	5 (62.5)
Adult outpatient	116	115 (99.1)	51	51(100.0)

The overall data accuracy for the health facilities in Ondo State was statistically higher than that of Ekiti State (70.1% versus 40.4%, p <0.001). The overall completeness of data reporting was 82.9% for Ondo State and 44.2% for Ekiti State (p <0.001). Almost all the health facilities in Ondo State (99.1%) and Ekiti State (96.2%) demonstrated consistency in terms of capturing the same RMNCAEH service items every month in the 12 months under review (p = 0.224). For Ekiti State, 94.2% of health facilities were verified to be submitting their required data to the Local Government level on timely basis monthly, compared to the significantly lower figure of 82.9% for health facilities in Ondo State (p = 0.048) (Table 6).

**Table 6:** Comparison of data quality elements between states.

Data quality elements	Ondo State n (%)	Ekiti State n (%)	p-value
Overall data accuracy			
Accurate	82 (70.1)	21 (40.4)	<0.001
Inaccurate	35 (29.9)	31 (59.6)	
Overall completeness of data reporting			
Complete	97 (82.9)	23 (44.2)	<0.001
Incomplete	20 (17.1)	29 (55.8)	
Data consistency			
Yes	116 (99.1)	50 (96.2)	0.224
No	1 (0.9)	2 (3.8)	
Timeliness in data reporting			
Yes	97 (82.9)	49 (94.2)	0.048
No	20 (17.1)	3 (5.8)	

### Processing, analysis, reporting and use of RMNCAEH+N data at the health facilities

Data processing procedures exist for RMNCAEH services rendered in 90.6% of health facilities in Ondo State and 86.5% of health facilities in Ekiti State, while 86.3% of facilities in Ondo State and 84.6% of facilities in Ekiti State calculates indicators for each facility catchment area, and 81.2% and 90.4% of facilities in Ondo and Ekiti States respectively compare data over time (monitoring trends over time). Virtually all the health facilities in the two states compile national HMIS health facility monthly summary form (NHMIS/HF/MSF) data on RMNCAEH services. Approximately three-quarters (74.4%) of facilities in Ondo State and two-thirds of facilities in Ekiti State (67.3%) had received feedback reports from the Local Government level regarding their submitted NHMIS/HF/MSF during the last three months preceding the study. Over 90% of health facilities in Ondo (92.3%) and Ekiti (90.4%) received quarterly/yearly feedback or any other report on NHMIS/HF/MSF data, which provides guidelines/ recommendations for actions for the improvement of the data system (Table 7).

**Table 7:** Processing, analysis, reporting and use of RMNCAEH+N data at the health facilities.

Variables	Ondo (N = 117) Yes (%)	Ekiti (N = 52) Yes (%)
***A*. *Processing*, *analysis and reporting of RMNCAEH+N data at the health facilities***		
Data processing procedures or a tally sheet exists for RMNCAEH services rendered	106 (90.6)	45 (86.5)
Facility calculates indicators for each facility catchment area	101 (86.3)	44 (84.6)
Facility compares data over time (monitoring trends over time)	95 (81.2)	47 (90.4)
Facility compiles NHMIS/HF/MSF data on RMNCAEH services	116 (99.1)	52 (100.0)
LGA office sent a feedback report to the facility using NHMIS/HF/MSF information during the last three months preceding the survey	87 (74.4)	35 (67.3)
***B*. Use of information in available reports at the health facility**		
Availability of quarterly/yearly feedback or any other report on NHMIS/HF/MSF data which provides guidelines/ recommendations for actions	108 (92.3)	47 (90.4)
Decisions made based on management of NHMIS discussion, and discussion on NHMIS findings (n = 97 (Ondo); 50 (Ekiti)	90 (92.8)	48 (96.0)

Evidence on the existence of quality improvement teams was available in only 70.1% of Ondo State facilities and 78.0% of Ekiti State facilities (p = 0.338). Among facilities that reported the existence of QI teams, 72.1% in Ondo State and 79.2% in Ekiti State undertook regular review and use of data for decision-making on QI (p = 0.367). The proportion of QI teams that regularly extracted data, calculated, and visualised prioritized RMNCAEH+N quality indicators was significantly lower for Ondo State compared to Ekiti State (53.5% versus 77.1%, p = 0.007) (Table 8).

**Table 8:** Comparison of RMNCAEH+N data use by health facilities QI team between states.

	Ondo State n (%)	Ekiti State n (%)	p-value
Health facility has a QI team (n = 97 (Ondo); 50 (Ekiti)			
No	11 (11.3)	2 (4.0)	0.338
Yes, not observed	18 (18.6)	9 (18.0)	
Yes, observed	68 (70.1)	39 (78.0)	
QI team regularly review data and use it to make decisions on QI (n = 86 (Ondo); 48 (Ekiti)			
No	24 (27.9)	10 (20.8)	0.367
Yes	62 (72.1)	38 (79.2)	
Facility QI team regularly extract data, calculate, and visualize RMNCAEH +N quality indicators (n = 86 (Ondo); 48 (Ekiti)			
No	40 (46.5)	11 (22.9)	0.007
Yes	46 (53.5)	37 (77.1)	

## Discussion

This study assessed the performance of routine HMIS for reproductive, maternal, newborn, child and adolescent health (RMNCAH) in the context of quality of care in Ondo and Ekiti States, with significant focus on the quality of data and the use of data for decision-making on quality improvement. Quality of care has significant implications for the effectiveness of health care and the potential of the health system to improve health outcomes. Up to 15% of deaths in low- and middle-income countries are estimated to be due to poor quality of care [15]. Quality of care is particularly critical for RMNCAH as it is estimated that 6 in 10 neonatal conditions and half of maternal deaths in low-income countries are due to poor quality services [16]. A high performing HMIS plays a central role in driving evidence-based quality improvement initiatives and monitoring the performance and outcomes of quality-related initiatives. Nigeria is one of the 10 countries that established the Network for Improving Quality Care for Maternal, Newborn and Child Health in partnership with WHO in 2017 and has initiated some key actions in line with the focus of the Network but Nigeria’s RMNCAH’s statistics remain poor. Moreover, the evidence base is weak regarding the performance of RHMIS for RMNCAEH+N QoC data collection and use for decision-making in Nigeria. Our study covered elements of data collection, data quality, and data use regarding RMNCAH RHIS in Ondo and Ekiti States. Thus, this study filled an important knowledge gap.

Our findings showed that a high proportion of facilities in Ondo and Ekiti States have the relevant HMIS data collection registers and forms for different RMNCAEH+N services, stock out was a challenge in respect of some of the RMNCAEH+N service data collection tools and the experience of Ondo State and Ekiti State differs in that respect. More than a tenth of facilities in Ondo State, for example, experienced stock out of growth monitoring forms within the 12 months before the survey. Insufficient supplies and stock-out of HMIS registers and forms for RMNCAEH+N services is a challenge which has also been reported in previous studies [17, 18]. In a mixed-method study conducted in Ethiopia, for example, the shortage of data collection registers and forms featured prominently in the qualitative findings while shortage of some specific registers such as those for PNC and growth monitoring and promotion were also reported in the quantitative aspect of the study [17]. A previous study published in 2019 and which covered the six geopolitical zones of Nigeria reported that some NHIS forms and registers were non-available in several facilities, particularly the daily family planning attendance register [18]. Opportunities to collect vital information needed to improve RMNCAEH+N QoC are missed when data collection tools are not readily available. In situations where the registers are available but there are occasional stock-out of the monthly summary forms, as is the case in our study, there is the possibility that data collected at the health facility level may not be transmitted to the next higher level when there are no monthly summary forms to do so. Not transmitting the complete data collected at a lower level to the next higher level has huge implication on the quality of data available for decision-making. It is therefore very important that all stakeholders responsible for the provision and allocation of RMNCAEH+N registers and forms prioritize and ensure the availability of these materials in all health facilities at all times as these are important tools needed to collect the data which will be analysed, reported, and used for decision making to improve RMNCAEH+N QoC.

Our study provided findings for the three elements most often used in assessing data quality: accuracy, completeness and timeliness, as noted by Chen et al. [19]. In addition, our study also provided findings for a fourth element: data consistency, which is also used for assessing data quality [20]. We recorded an overall data accuracy of 70.1% for Ondo State and a significantly lower figure of 40.4% for Ekiti State. In comparison to our study findings for data accuracy, studies conducted within and outside Nigeria have reported varying performance of RHIS for RMNCAEH + N. Belay et al. in Ethiopia, for example, reported data accuracy of 41.4% for three RMNCAEH+N data elements assessed in their study [21] while Sharma et al. in an Indian study found over-reporting which ranged from 1.4% to 6.0% across 14 maternal and child health data elements when the numerical difference between the monthly figures in the Auxiliary Nurse Midwives (ANMs) record registers and the figures in the monthly reports submitted by ANMs to the Primary Health Centre was determined [22]. Under-reporting and over-reporting of health facility immunization data were also reported by Ogbuabor et al. in a study conducted in South-Eastern Nigeria [23]. The differences in how data accuracy is measured in each of the studies makes comparability of the study findings a bit difficult. Hence, the need for studies to assess data accuracy and other data quality elements using unified and standard methods cannot be overemphasized. Our study showed considerable variation in data accuracy for different service elements and significant difference between the performance of health facilities in Ondo and Ekiti States. At least 90% of health facilities in Ondo State demonstrated data accuracy for nine out of the 10 service elements while the health facilities in Ekiti State recorded similar achievement with respect to only three service elements. Although the performance of health facilities in Ondo State for data accuracy could be considered to be better than the performance of health facilities in Ekiti State, the odds of poor decision making and misallocation of scarce resources when data accuracy is anything short of 100% is very high.

Previous studies conducted in Ethiopia, Ghana, and Nigeria documented routine data completeness ranging from 60% to 92.9% [21, 24–26], and our finding of overall data completeness of 82.9% for Ondo State falls within the reported range while the completeness of 44.2% for Ekiti State falls outside the reported range. We recorded 99.1% for health facilities in Ondo State and 96.2% for facilities in Ekiti State in terms of data consistency, and 82.9% for Ondo State and 94.2% for Ekiti State for timeliness of data submission. These findings are comparable to what other authors have reported in the literature [24, 26]: Shama et al., for example, reported a figure of 93.7% for data timeliness in an Ethiopian context [24]. Similar to data accuracy, any data quality element: completeness, consistency, or timeliness, short of 100% is not the best for proper decision making.

As Nigeria is currently experiencing serious human resource for health shortage [27, 28], a possible explanation for the inaccurate, incomplete, inconsistent, and untimely data for RMNCAEH+N services recorded in Ondo and Ekiti States could be due to the use of unqualified staff with lack of requisite HMIS knowledge and skills to replace the HMIS professionals who have left the employ of the public health sector. Another possible explanation, which can also be linked to the manpower shortage, is staff work overload and fatigue which can cause errors in data reporting. Other possible reason could be due to unavailability of resources needed for data collection and transmission. When the standard data collection tools are unavailable, the concerned staff resort to improvisation in the interim. Errors can occur in the process of transferring data form the interim measure to the acceptable tool anytime the standard tools are available. Training and re-training can help solve the problem in the interim. However, a longer lasting recommendation to solving this problem of data inaccuracy, incompleteness, inconsistency, and untimeliness is that concerned stakeholders in the two states should dig further to determine the root cause of the problem and solving the problem at its roots.

In addition to data collection, we also assessed the performance of routine health information system (RHIS) data use. Not all health facilities visited in our study sites processed, analysed, reported, or used the RHIS data they collect. As documented in a previous study, a possible underlying factor for the problem of not reporting or using collected data noted by our study could be lack of commitment on the part of heads of the facilities to constitute a quality improvement team and drive the quality improvement agenda at the health facility level [29]. Another possible explanation from the same Ugandan study is the high turnover rate of staff [29]. Nigeria had been and is still experiencing serious human resources for health shortage [27, 28]. It is very possible that health workers who have been trained on QI are among those leaving the health workforce in Ondo and Ekiti States. It is also very possible that the human resource for health still in the employ of both states are overworked as noted earlier and cannot combine their primary assignment with their quality improvement role. Other possible explanations for our study findings could be that the financial resources and material resources to effect the desired quality of care change are not available which makes the quality improvement team handicapped. The root causes of why the data collected are not been utilized need to be identified and tackled, using a qualitative approach, if data collection and process are to translate to improved quality of care.

The coverage of our study is two neighbouring states in southwest Nigeria–Ondo and Ekiti States. It is important to note that the Nigeria Demographic and Health Survey that served as the evidence base for the selection of these two states based on their poor RMNCAH performance is the most current for the country, it was almost 5 years old at the time of this study. Also, it is important to note that the south-west region has about the best RMNCAEH+N data of all Nigerian zones, and significantly better than each of the three northern zones [3]. Thus, caution should be exercised in generalizing our study findings to the whole of Nigeria for decision-making as findings from these two states are not likely to be representative of Nigeria as a whole. Furthermore, we purposively selected two of the three senatorial zones in each of the two states for safety reasons because of the high-security threat in the zones excluded from the study. Given the high potential of such security challenges to negatively affect and disrupt health services and the functioning of RHMIS, the findings from our study may not also be directly applicable across the entire Ondo and Ekiti States. More representative studies capturing the six Nigerian geopolitical zones and the entire senatorial districts in selected states are recommended in the future. Despite these limitations, our study adds significantly to the body of knowledge on performance of the RHIS and RMNCAEH+N information system in Nigeria.

Overall, the performance of the RHIS for RMNCAEH+N in Ondo and Ekiti States is fair-to-good for most of the elements assessed in the study, but there are also significant differences in performance between the two states despite their shared culture, political history, and developmental trajectory. Ondo State performed significantly better in terms of data accuracy and data completeness, while Ekiti State has a borderline significantly better performance than Ondo State in terms of timeliness of data submission and engagement of the QI teams in regularly extracting data, calculating, and visualising prioritized indicators. Thus, recent and current factors in the health system of the two states are likely to account for the different performances recorded, and relevant interventions can lead to significantly improved performance. Our findings provide evidence regarding the areas of strength as well as the areas of deficiencies for the two states. The areas of strength call for continuous maintenance of efforts to sustain and even improve the performance, while the areas of deficiencies bring attention to elements where critical review and urgent actions are needed to improve the performance of HMIS as a tool for changing the narratives of poor quality of RMNCAEH+N services in Nigeria. As the World Health Organization has aptly noted, “data availability does not automatically translate into availability of the quality data needed for policy, planning, and patient health care [30].” As a study in two states in northern states in Nigeria showed, the implementation of continuous quality improvement has the potential to improve the utilization of data to drive improved quality of services [31]. In this respect, greater attention needs to be given to the capacity, functionality and effectiveness of health facility QI teams whose job description will include the use of all data generated at the health facility level, including RMNCAEH QoC data, to make informed decisions that would actively drive quality improvements.

## Conclusion

Our findings suggest that Ondo and Ekiti States performed fairly well with respect to routine RMNACEH+N QOC data. As such, efforts are needed to address the areas of poor performance as well as to sustain continued high-level performance in areas that the states have done well. Consequently, the findings from this study provide a strong platform for initiating evidence-based advocacy to drive the required efforts.
